# Mitochondria in Embryogenesis: An Organellogenesis Perspective

**DOI:** 10.3389/fcell.2019.00282

**Published:** 2019-11-22

**Authors:** Yoan Arribat, Dogan Grepper, Sylviane Lagarrigue, Joy Richard, Mélanie Gachet, Philipp Gut, Francesca Amati

**Affiliations:** ^1^Aging and Muscle Metabolism Lab, Department of Physiology & Institute of Sport Sciences, School of Biology and Medicine, University of Lausanne, Lausanne, Switzerland; ^2^Nestlé Research, Nestlé Institute of Health Sciences, Lausanne, Switzerland

**Keywords:** electron transport chain supercomplexes, mitochondria fission, morphogenes, myomaker, somite, sonic hedgehog, zebrafish

## Abstract

Organogenesis is well characterized in vertebrates. However, the anatomical and functional development of intracellular compartments during this phase of development remains unknown. Taking an organellogenesis point of view, we characterize the spatiotemporal adaptations of the mitochondrial network during zebrafish embryogenesis. Using state of the art microscopy approaches, we find that mitochondrial network follows three distinct distribution patterns during embryonic development. Despite of this constant morphological change of the mitochondrial network, electron transport chain supercomplexes occur at early stages of embryonic development and conserve a stable organization throughout development. The remodeling of the mitochondrial network and the conservation of its structural components go hand-in-hand with somite maturation; for example, genetic disruption of myoblast fusion impairs mitochondrial network maturation. Reciprocally, mitochondria quality represents a key factor to determine embryonic progression. Alteration of mitochondrial polarization and electron transport chain halts embryonic development in a reversible manner suggesting developmental checkpoints that depend on mitochondrial integrity. Our findings establish the subtle dialogue and co-dependence between organogenesis and mitochondria in early vertebrate development. They also suggest the importance of adopting subcellular perspectives to understand organelle-organ communications during embryogenesis.

## Introduction

Embryogenesis follows an evolutionary conserved stereotypical sequence of events leading to the formation of functional tissues and organs. Signaling pathways involved in these processes have been extensively characterized, and include the morphogenes Sonic Hedgehog (Shh) and Bone Morphogenic Protein (BMP), among many others (Maurya et al., 2011). Although invertebrate development shares commonalities with vertebrates, neuromuscular structuration in the latter follows a unique patterning during early developmental stages (Lewis et al., 1999; Lewis, 2006). Somitogenesis encompasses separation of somites, epithelialization, specification along the antero-posterior axis, and differentiation into sclerotome and dermomyotome (Stickney et al., 2000; Windner et al., 2012; Musumeci et al., 2015). Muscles derive from myotome cells, with common steps in embryonic and adult myogenesis, including myoblasts proliferation, fusion in multinucleated cells and final maturation into myofibers (Paululat et al., 1999). The mechanisms conducting cell fate upon embryogenesis have been extensively described, but one of the major challenges facing the field is to understand embryogenesis from the organelles point of view, and particularly regarding mitochondria.

In recent years, our understanding in mitochondria biology has drastically increased. Mitochondria are key to supply the required energy for cellular homeostasis through the electron transport chain (ETC), which builds-up the necessary proton gradient to produce ATP. Following the *plasticity model*, free moving ETC complexes transiently form super-assembled structures called supercomplexes (SCs) mostly composed of complex (C) I, CIII and CIV subunits (Lapuente-Brun et al., 2013; Acin-Perez and Enriquez, 2014). SCs enhance the stability of individual ETC complexes (Acin-Perez et al., 2008), increase electron transport efficiency and consequently limit reactive oxygen species (Genova and Lenaz, 2015; Cogliati et al., 2016). SCs have been reported in mature tissues in human, rodents and zebrafish (Lapuente-Brun et al., 2013; Greggio et al., 2017; Parisi et al., 2018). Their timing of appearance in embryogenesis is yet unknown.

To face changing energetic demands in adult tissues, the mitochondrial network is highly dynamic with the capacity to generate new mitochondria through biogenesis, modify the network through fusion and fission, and clear damaged mitochondria through mitophagy (Yu and Pekkurnaz, 2018). While these mechanisms have been reported in several tissues and post-developmental conditions, little is known on the importance of mitochondria dynamics in developing tissues, and particularly in embryogenesis.

Maternal mitochondria supply the required energy for oocyte viability (Wilding et al., 2009). Maternal supply ensures functional mitochondria until embryonic mitochondria take over (Artuso et al., 2012). This new mitochondrial network faces continuous challenges. Indeed, the embryo is submitted to successive series of changes requiring permanent adaptations of mitochondria. The growth of the mitochondrial population in the embryo is discontinuous with a burst during oogenesis followed by an arrest after fertilization during cleavage stages until mitochondrial replication resumes after gastrulation (Dumollard et al., 2006, 2007). While mitochondria adaptations and roles in energy production, calcium homeostasis, oxidative stress and apoptosis have been extensively described in the very first steps of embryogenesis (Bavister and Squirrell, 2000; Squirrell et al., 2003; Lima et al., 2018), observations are limited after the blastocyst stage due to internal development after implantation in mammals. We hypothesized that the mitochondrial network plays a crucial role after gastrulation in order to respond to the rhythm of organ maturation and adapt in unison. Our main objective was to describe the timing and key steps of mitochondrial adaptation during post-gastrulation embryogenesis and cell differentiation. Further, we explored the influence of mitochondrial activity on embryogenesis progression rate. Finally, we questioned the existence of a conductor synchronizing somitogenesis with organellogenesis.

## Results

### Zebrafish, a Relevant Model to Follow Mitochondrial Pattern of Change in Embryonic Development

In mammals, internal fecundation and development limit observations of subcellular organelles in post implantation stages. Rodent models allow observations at key stages but are limited by the inability to follow live embryogenesis (Strittmatter, 1963; Mackler et al., 1971; Bavister and Squirrell, 2000; Wilding et al., 2001). Due to stereotypical and evolutionarily conserved processes in vertebrate early stages of life; chicken, xenopus and fish are useful models to overcome these limitations (Webb and Smith, 1977; Kim et al., 2008). Zebrafish embryos represent a perfect model to characterize the developmental machinery because of their transparence and speed of embryogenesis. Indeed, a new pair of somite is formed each 30 min in zebrafish, which is four times faster than in mice (Resende et al., 2014).

**FIGURE 1:**
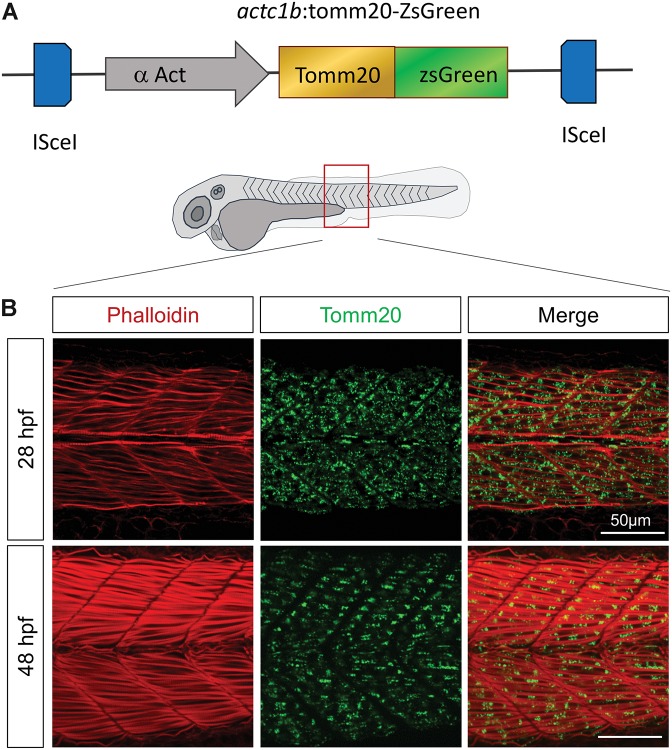
Tomm20-zsGreen transgenic zebrafish represent a relevant model to decipher mitochondrial modifications during embryogenesis. **(A)** Transgenic construct expressing zebrafish Tomm20 under the control of α Actin muscle specific promoter. **(B)** Confocal imaging of mitochondrial network (Tomm20, green) at the limit of yolk extension corresponding to somites 15–17, counterstained with Phalloidin (red) at 28 and 48 hpf. See also Supplementary Figure S1.

Given that endogenous components of mitochondria fluctuate during embryogenesis (Artuso et al., 2012), expressing a stable exogenous reporter embodies a relevant strategy to follow in real time mitochondria pattern of change during the successive steps of development. To this end, we expressed a GFP-tagged transgene of zebrafish *tomm20*, which encodes an outer mitochondrial membrane protein, under the control of muscle specific α actin promotor (*actc1b:tomm20-ZsGreen;cryaa:TdTomato^*nei*007^*; hereafter named *actc1b*:tomm20-ZsGreen) (Figure 1A). This model allows the observation of the mitochondrial network reorganization between the beginning of somitogenesis (11 h post-fertilization; hpf) and the end of embryogenesis (48 hpf), which is concomitant with the maturation of muscle fibers (Figure 1B and Supplementary Figure S1). We confirmed these observations in an equivalent transgenic model in which the GFP reporter was fused with MLS mitochondrial targeting sequence (Kim et al., 2008; Zhang et al., 2015) (data not shown).

### Mitochondria Network Follows Three Patterns of Change in Embryogenesis

**FIGURE 2:**
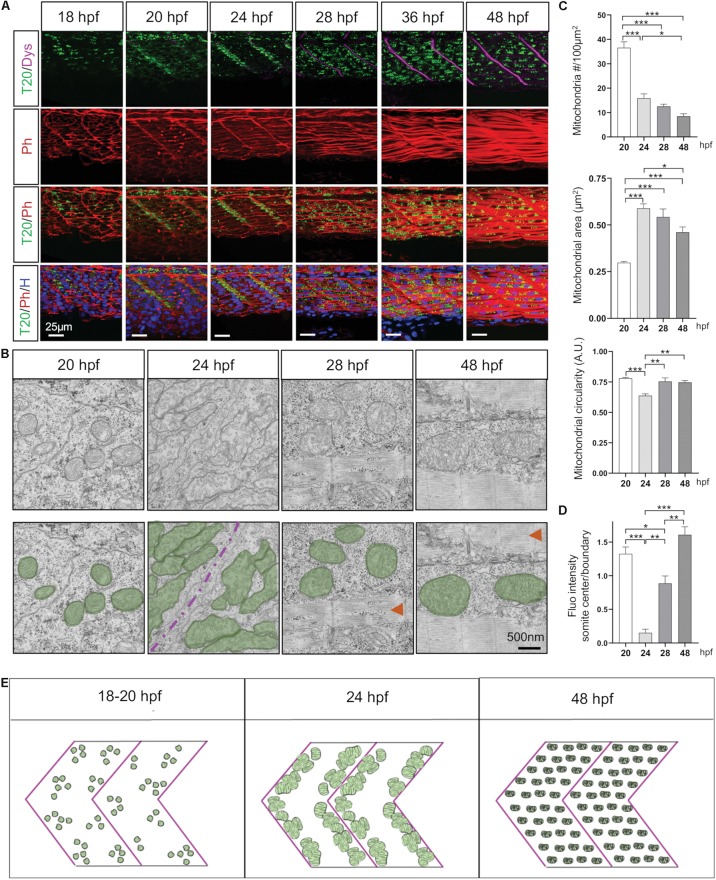
Mitochondria network adaptation follows three patterns of change through embryogenesis. **(A)** Confocal imaging of mitochondrial network (Tomm20, T20; green) counterstained with Phalloidin (Ph; red), Dystrophin (Dys; Magenta) and Hoescht (H; blue) at 18, 20, 24, 28, 36, and 48 hpf. Pictures taken at the limit of yolk extension (somites 15–17). **(B)** Electron micrographs of longitudinal sections at 20, 24, 28, and 48 hpf. Green overlays highlight mitochondria, red arrows indicate sarcomeric structures, magenta line is the separation between two somites. **(C)** Quantitative analyses of mitochondrial number, area and circularity at 20, 24, 28, and 48 hpf (*n* = 6 micrograph areas of 200 × 200 μm^2^ analyzed per group). **(D)** Quantification of Tomm20-zsGreen fluorescence ratio between somite center and boundary region at 20, 24, 28, and 48 hpf (*n* = 6 fish per group, 3 images analyzed per fish). **(E)** Cartoon depicts three distinct patterns presented by the mitochondrial network through embryogenesis. Bars are mean ± SEM. ^∗^*P* < 0.05, ^∗∗^*P* < 0.01, ^∗∗∗^*P* < 0.001, one way repeated measures ANOVA with Tukey HSD *post hoc* test. See also Supplementary Figures S2–S5 and Supplementary Video S1.

To explore mitochondrial network modifications throughout all steps of embryogenesis, we used state-of-the-art imaging techniques including live time-lapse recording on anesthetized embryos with a double illumination inverted light sheet microscope (ILS1 Live, Viventis Microscopy Sàrl, EPFL, Lausanne, Switzerland) (Supplementary Video S1). Mitochondria follow three different distribution patterns (Figure 2 and Supplementary Figure S2). At 14 hpf (10-somite), mitochondria expressing the reporter Tomm20-zsGreen start to appear. At 18 hpf (18-somite), mitochondria are homogeneously distributed across myotomes (Figure 2A). From 20 hpf (21-somite), we observe a distinct evolution of mitochondria in fast and slow fibers. In slow fibers, which are located at the periphery, mitochondria are diffusely spread and conserve a homogenous pattern throughout the rest of embryo development (Supplementary Figure S3). In fast fibers, concomitantly to fiber differentiation and elongation, mitochondria redistribute close to somite boundaries (Figure 2A and Supplementary Figure S2) with an average of 36.54 mitochondria per 100 μm^2^ and a mean mitochondrial area of 0.269 μm^2^ (Figure 2C). At 24 hpf (prim-5) mitochondria fully relocate to somite boundaries (Figures 2A,B,D). At this stage, mitochondria are elongated, their mean area increases by twofolds and mitochondrial number per 100 μm^2^ decreases which is consistent with their concentration at the boundaries (Figure 2C). At 28 hpf, mitochondria spread again across the myotome concomitantly to muscle fibers thickening at 36 hpf (prim-25) and 48 hpf (Supplementary Figure S2). While mitochondria mean area remains stable, mitochondria number decreases in accord to somite elongation. Staining wild type muscle with antibodies against MTCO1, an endogenous component of CIV, confirmed these three distinct and successive distribution patterns revealed by the reporter line (Supplementary Figure S4).

In summary, mitochondria patterning follows a systematic time-course evolution within each somite in parallel to myofiber maturation (Figure 2E). First, small and numerous mitochondria are present in myoblasts. As myoblasts fuse, mitochondria are accumulated at somite boundaries. Finally, mitochondria spread ensuring their redistribution through mature myofibers at the end of embryogenesis (Supplementary Figure S1). Importantly, this patterning follows the rostro-caudal coupling of somitogenesis and axis elongation (Supplementary Figure S5 and Supplementary Video S1).

### Electron Transport Chain Supercomplexes Appear Early in Embryogenesis

**FIGURE 3:**
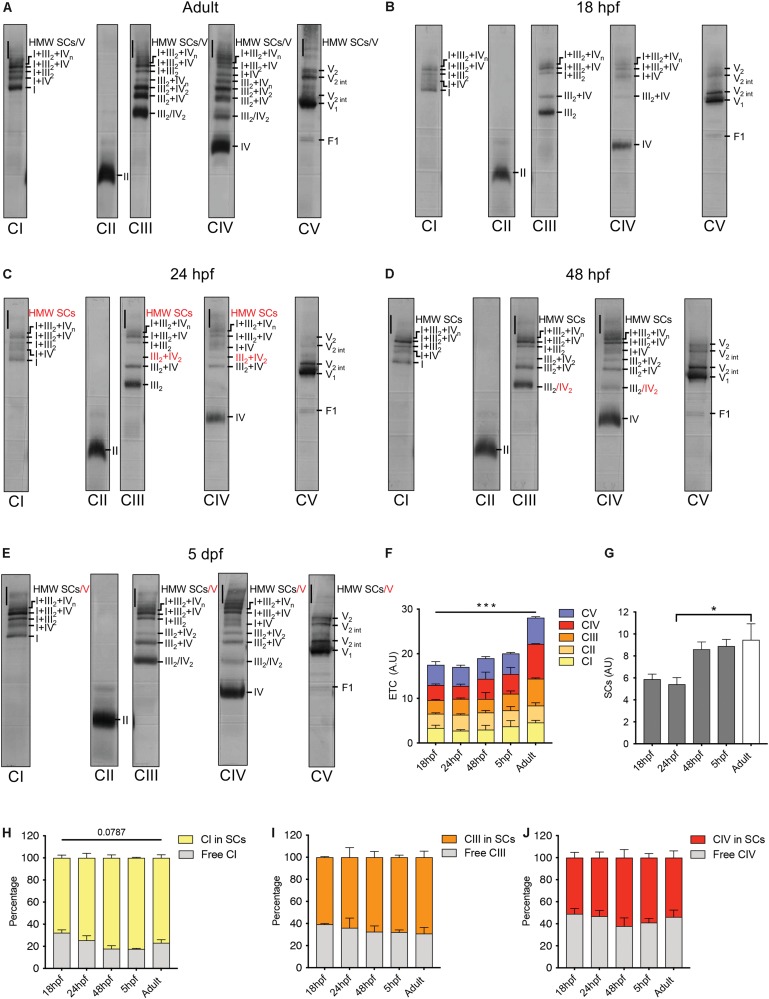
Electron transport chain supercomplexes are qualitatively stable throughout zebrafish development. **(A–E)** Representative BN-PAGE experiment with mitochondrial extracts from adult fish (6 months), 18, 24, 48 hpf, and 5 dpf. Specific antibodies against individual ETC complexes were used separately. Red numbers correspond to new bands appearing at particular time points. HMW is high molecular weight, int is intermediate. **(F)** Quantification of individual ETC complexes. Each value represents the signal on the immunoblot for each ETC complex (*n* = 2, each n with 250 embryos per group). **(G)** Quantification of supercomplexes (SCs). Each value represents the sum of all SCs bands including III_2_ + IV, III_2_ + IV_2_, I + III_2_, I + IV, I + III_2_ + IV, I + III_2_ + IV_n_ and HMW SCs (*n* = 2, each n with 250 embryos per group). **(H–J)** Quantification of the percentage of CI, CIII, and CIV in free form and in SCs (*n* = 2, each n with 250 embryos per condition). Bars are mean ± SEM. ^∗^*P* < 0.05, ^∗∗∗^*P* < 0.001, two way repeated measures ANOVA (line is effect of time) or one way repeated measures ANOVA with Tukey HSD *post hoc* comparison (bracket). See also Supplementary Figure S6.

To explore how the ETC faces the challenge of organogenesis from a structural point of view, we performed blue native polyacrylamide gel electrophoresis (BN-PAGE) of mitochondrial extracts from 18 hpf, 24 hpf, 48 hpf, 5 days post-fertilization (dpf) and adult fish. We first labeled specific SCs of adult zebrafish (Figure 3A) following the nomenclature previously used (Schagger, 2002; Schagger et al., 2004; Sun et al., 2016; Wu et al., 2016; Greggio et al., 2017). Consistent with former reports in other species (Acin-Perez et al., 2008; Greggio et al., 2017), CII was not associated to SCs. CI, CIII and CIV were present in both free and superassembled forms. CV was evidenced in mono and dimeric structures as well as in intermediate forms (Wittig et al., 2008). The adult pattern was used as reference for the labeling of all other stages (Figures 3B–E and Supplementary Figure S6). SCs are already present in mitochondria at 18 hpf, followed by the progressive appearance in the successive developmental stages of specific bands among which SC III_2_ + IV_2_ and high molecular weight (HMW) SCs (Figures 3B–E). While no interaction effect is detected with a two way repeated measures ANOVA, there is a significant effect of time on the distribution of each ETC during zebrafish development (Figure 3F). The overall content of SCs follows the same pattern with a significant effect of time explained by the difference between 18 hpf and adult (one way repeated measures ANOVA, Figure 3G). We did not evidence significant differences across time for the relative participation of CI, CIII and CIV in SCs or the free forms (Figures 3H–J). Taken together, these results demonstrate that SCs are present during embryogenesis with increments over time and that their overall content is relatively stable from the end of embryogenesis to adulthood.

### Mitochondria Dynamics Support Mitochondria Distribution Patterns

**FIGURE 4:**
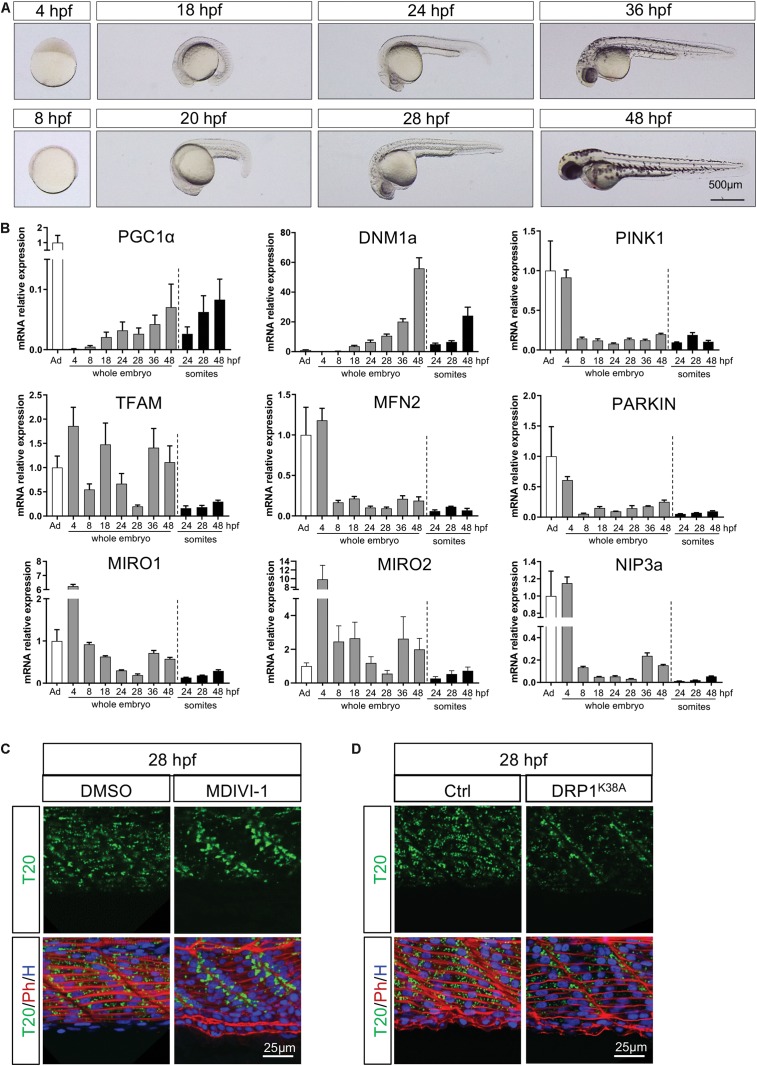
Temporal configuration of mitochondrial biogenesis, fusion, fission, mitophagy and transport through embryogenesis. **(A)** Representative pictures illustrate the different stages of zebrafish development used for quantitative RT-PCR. **(B)** Gene expression of key mitochondrial actors in whole embryos or dissected somites. Values are estimated using the 2^–ΔΔCT^ method. Gene expression is normalized to 18S and compared to adult zebrafish muscle (bars are mean ± SEM). **(C)** Confocal imaging of mitochondrial network (Tomm20, T20; green) counterstained with Phalloidin (Ph; red) and Hoescht (H; blue) at 28 hpf in presence or absence of the fission inhibitor MDIVI-1 incubated since 24 hpf. **(D)** Confocal imaging of mitochondrial network (Tomm20, T20; green) counterstained with Phalloidin (Ph; red) and Hoescht (H; blue) at 28 hpf in embryos injected with mRNA encoding for the dominant negative DRP1 or with a mock at the one-cell stage zygote.

To identify the specific pathways involved in the three successive mitochondria patterns of change, we measured mRNA encoding key actors of mitochondrial dynamics at different developmental stages (Figures 4A,B and Supplementary Table S1). Statistical analyses were performed with one way repeated measures ANOVA to evaluate the effect of time. To determine regional specificity, we compared transcripts in dissected somites at 24, 28, and 48 hpf to transcripts in whole embryo at the same time points with two way ANOVA. Across most targets, maternal supply is apparent at 4 hpf with residual levels at 8 hpf (Figure 4B).

Mitochondrial biogenesis depends upon nuclear encoding transcription factors, mainly Mitochondrial Transcription Factor A (TFAM) which responds to Peroxisome Proliferator Activated Receptor Gamma Co-activator 1 alpha (PGC1α). PGC1α follows a gradual increase in whole embryos, mostly driven by somites, until the end of embryogenesis (significant effect of time). After the decrease in maternal supply, TFAM expression is submitted to two consecutive waves at 18 hpf and from 36 hpf in the whole embryos (significant effect of time), with lower levels in somites (significant regional difference). These findings suggest two bursts of biogenesis upon embryo maturation. The first transient peak is ensured by TFAM in the whole embryo and immediately precedes mitochondria accumulation at the boundaries at 24 hpf. The second is supported by the increase of PGC1α from 36 hpf until the end of embryogenesis at 48 hpf. While the second peak of mitochondrial biogenesis is also supported by TFAM transcripts in the whole embryo, it is not the case in somites (Figure 4B).

The balance between fusion and fission is an important mechanism participating in the control of mitochondria number and size. Transcripts of Mitofusin 2 (MFN2), one of the highly conserved GTPases responsible for fusion, are relatively low and stable across development time-points (Figure 4B). Transcripts of Dynamin 1 isoform A (DNM1a), main driver of fission, increase gradually in whole embryos (significant effect of time) with an exponential burst at 48 hpf that is paralleled in somites (interaction effect explained by the significant regional difference at 48 hpf, Figure 4B). To confirm the role of fission at the end of embryogenesis, we delivered 50 μM of MDIVI-1, a specific inhibitor of DNM1 GTPase activity, to 24 hpf embryos (Cassidy-Stone et al., 2008; Manczak et al., 2019). In absence of fission, mitochondria do not spread across the myotome at 28 hpf and remain stacked at the somite edges (Figure 4C). Given the debated effect of MDIVI-1 on CI efficiency (Bordt et al., 2017; Smith and Gallo, 2017), we complemented this observation overexpressing the dominant negative version of DNM1 (Smirnova et al., 1998). The injection of mRNA encoding the mutant DRP1^K38A^ in one-cell zygote confirms the importance of fission to ensure mitochondrial spreading across the myotome observed after 24 hpf (Figure 4D).

As mitochondria need active transport, we measured transcripts of Mitochondrial Rho GTPase 1 and 2 (MIRO1 and 2), two important actors in mitochondrial trafficking. Both MIRO1 and MIRO2 exhibit a high maternal supply and a gradual decrease until 28 hpf (significant effect of time), followed by a burst of relative expression at 36 and 48 hpf in whole embryos (Figure 4B). The fact that these bursts are not observed in somites suggests a high level of expression in the head and brain at 36 and 48 hpf (significant interaction for MIRO1 and significant regional difference for MIRO2). This observation is coherent with the very dynamic mitochondrial transport described in posterior lateral line neurons in 30 and 48 hpf embryos (Mandal et al., 2018).

Mitochondrial clearance is mediated by the autophagy lysosomal machinery through the P-ten Induced Putative Kinase 1 (PINK1)/PARKIN cascade or BCL2/adenovirus E1B19KDa Protein/interacting protein 3 (BNIP3). In zebrafish, the functional homolog of BNIP3 is NIP3a (Feng et al., 2011). For all of these transcripts, we observe a significant effect of time explained by the drop between maternal supply at 4 and 8 hpf (Figure 4B). This mitophagy peak mediated by maternal supply may participate in the degradation of paternal organelle has described immediately after the fertilization (Al Rawi et al., 2011, 2012; Rojansky et al., 2016). From 8 hpf until the end of embryogenesis, we witness low and stable levels of PINK1, PARKIN and NIP3a expression, especially in somites (interaction effect for PINK1 and NIP3a, significant regional difference for PARKIN). Mitophagy does not seem required during somitogenesis, which goes well with the needed increase in mitochondrial mass.

### Mitochondria Spreading From Somite Boundary Is Not Controlled by Neuronal Activity or Muscle Contraction Rate

**FIGURE 5:**
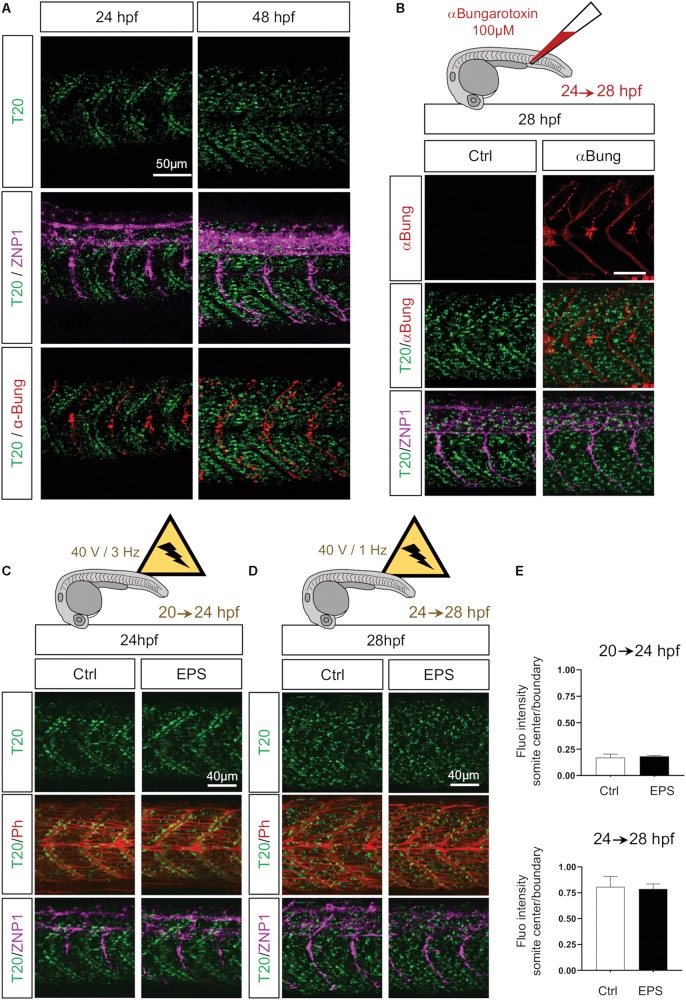
Neuronal stimulation and muscle contractions do not mediate mitochondrial patterning. **(A)** Confocal imaging of mitochondrial network (Tomm20, T20; green) counterstained with a marker for neuromuscular junctions αBungarotoxin (αBung; red) and a marker for primary motor neurons (ZNP1; magenta) at 24 and 48 hpf. While T20 signal corresponds to a unique confocal stack, αBung and ZNP1 represent the Z project. **(B)** Confocal imaging of mitochondrial network (Tomm20, T20; green) counterstained with αBungarotoxin (αBung; red) and primary motor neurons (ZNP1; magenta) at 28 hpf, with or without intravenous injection of αBungarotoxin performed at 24 hpf. **(C)** Confocal imaging of mitochondrial network (Tomm20, T20; green) counterstained with Phalloidin (Ph; red) at 24 hpf, with or without electrical pulse stimulation (EPS) applied at 20 hpf. **(D)** Confocal imaging of mitochondrial network (Tomm20, T20; green) counterstained with Phalloidin (Phall; red) at 28 hpf, with or without electrical pulse stimulation (EPS) applied at 24 hpf. **(E)** Quantification of Tomm20-zsGreen fluorescence ratio between somite center and boundary region at 24 and 28 hpf after 4 h of EPS (n = 6 fish per group, 3 images analyzed per fish).

Motor axon maturation takes place between 24 and 28 hpf (Stickney et al., 2000). This timing, which is simultaneous to the observed spread of mitochondria from somite boundaries across the myotome, interrogates whether the raise in neuromuscular junction plays a role in mitochondrial distribution. Indeed, from 22 hpf, caudal primary motor neurons (CaP) axons start to reach their distinct location in the rostral region generating acetylcholine synapses between primary motor axons and muscle fibers, thus forming neuromuscular junctions along the rostro-caudal maturation axis. By co-staining primary motor neurons with anti-Synaptotagmin2 (ZNP1) and neuromuscular junctions with the potent agonist of nicotinic acetylcholine receptor, αBungarotoxin, we confirmed that, at 24 hpf, CaP motor neurons form a network of synapses en passant along each somite (Panzer et al., 2005) with their axons extending ventrally through the median axis of the myotome (Figure 5A). At 48 hpf the maturation of the neuromuscular junctions is complete as CaP axons reach the ventral edge, turn dorsally and laterally to project over the lateral surface of the axial muscles (Kuwada et al., 1990) (Figure 5A). To measure the impact of CaP axons (i.e., neuronal stimulation) on mitochondrial spreading, we blocked neuromuscular synapses injecting 4 nl of 100 μM α Bungarotoxin in the bloodstream of 24 hpf embryos. This irreversible ligand of the nicotinic acetylcholine receptor results in a complete paralysis of the embryo compared to the injection of mock solution. Despite the persistent presence of α Bungarotoxin at 28 hpf, mitochondria spreading is unaffected (Figure 5B). These results show that primary motor neurons do not influence mitochondria patterns of change in myocytes.

We next questioned whether contractions could modulate mitochondrial network patterns of change. Indeed, zebrafish embryos exhibit contractions starting at 17 hpf independently from primary motor neuron stimulation (Saint-Amant and Drapeau, 1998; Hirata et al., 2009). These contractions seem to participate to muscle fibers maturation notably through calcium signaling (Brennan et al., 2005). Here, we electrically stimulated dechorionated embryos, from 20 to 24 hpf and from 24 to 28 hpf, to obtain twofold increases from endogenous basal contractions (Saint-Amant and Drapeau, 1998). Pulses of 2.0 ms, with 40 V and 3 Hz from 20 to 24 hpf do not affect the accumulation of mitochondria at the boundaries (Figures 5C,E). Pulses of 2.0 ms, with 40 V and 1 Hz from 24 to 28 hpf do not disturb the redistribution of mitochondria from the edges to the center of the myotome (Figures 5D,E). Thus, higher rates of contractions were unable to modify mitochondria distribution (Figure 5E). These observations exclude a control of muscle activity *per se* on organelle spatio-temporal adaptation.

### Maturation of Mitochondrial Network Is Contingent on Myoblast Fusion

**FIGURE 6:**
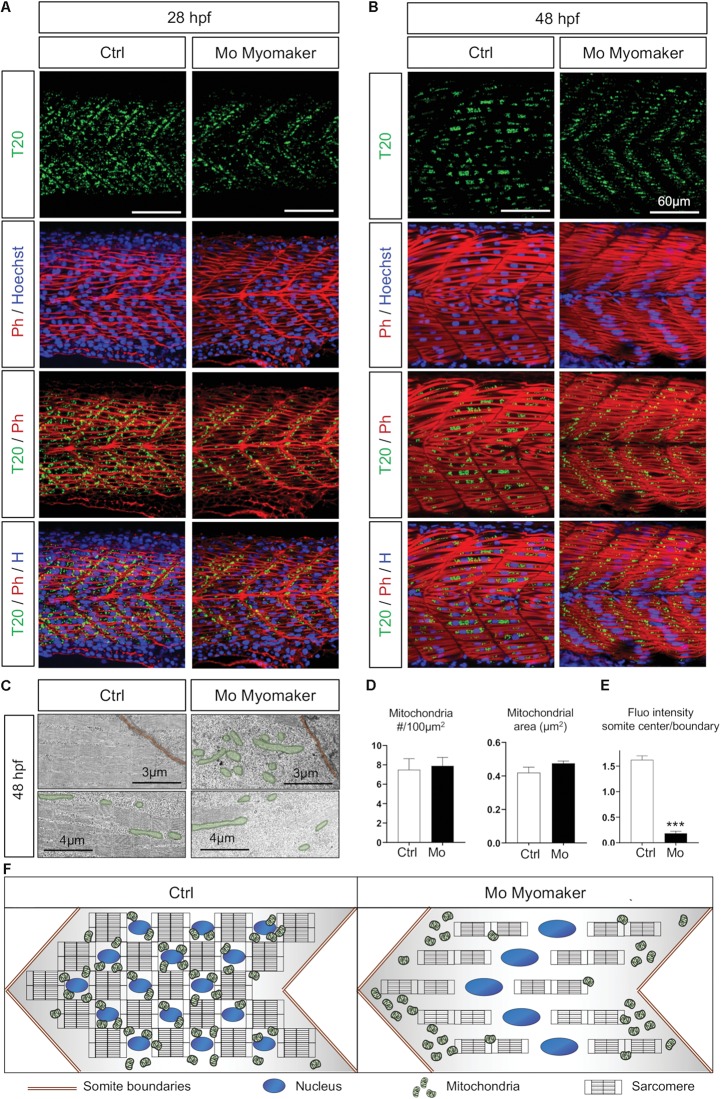
Mitochondrial network maturation is conditional to myoblast fusion. **(A,B)** Confocal imaging of mitochondrial network (Tomm20, T20; green) counterstained with Phalloidin (Ph; red) and Hoescht (H; blue) at 28 hpf **(A)** and 48 hpf **(B)**; control (Ctrl, mock injection) or Myomaker-targeting Morpholinos (Mo Myomaker). **(C)** Electron micrographs of longitudinal sections at 48 hpf in control and Myomaker depleted embryos. Top images are close to boundaries, bottom images are at the somite center. **(D)** Quantification of mitochondrial number and area at 48 hpf from longitudinal electron micrographs (*n* = 6 micrographs for Ctrl, *n* = 7 micrographs for Mo). **(E)** Quantification of Tomm20-zsGreen fluorescence ratio between somite center and boundary region 48 hpf (*n* = 4 fish per group, 3 images analyzed per fish). **(F)** Cartoon representing somite structure and mitochondrial network at 48 hpf in control and Myomaker depletion. Bars are mean ± SEM. ^∗∗∗^*P* < 0.001, unpaired *T*-test.

As the synchronization between mitochondria and myotome development is independent from neuromuscular influence, we next hypothesized a direct link between myocyte maturation and mitochondrial patterning in embryos. Indeed, the burst of mitochondrial biogenesis observed at 24 hpf is concurrent to myoblast fusion. To decipher the association between these events, we modulated myofiber maturation through depletion of Myomaker (Tmem8c) (Figures 6A,B). This transmembrane protein, highly conserved in vertebrates, is key for membrane merging and myoblasts fusion upon muscle maturation (Millay et al., 2013; Landemaine et al., 2014). Injection of 0.5 pmol of AUG Morpholinos targeting *myomaker* expression during zebrafish somitogenesis lead to mononucleated myocytes at 48 hpf instead of syncytial muscle fibers (Figure 6B) (Landemaine et al., 2014; Zhang and Roy, 2017). Depletion of Myomaker not only affects myocyte fate, but also alters mitochondria spreading from somite boundaries preventing their distribution across the myotome at 28 hpf (Figure 6A). Electron microscopy confirmed that Myomaker-depleted embryos exhibit an abnormal mitochondrial pattern with accumulations close to somite edges and reductions around nuclei at 48 hpf (Figure 6C). At 48 hpf, these defects persist with mitochondria stacked to the edges of the mononucleated myofibers (Figure 6B) and reduced numbers of mitochondria close to the sarcomeres (Figure 6C). Quantitative analyses performed at 48 hpf show no impact on mitochondria number and mean area (Figure 6D) or on mitochondrial circularity (data not shown). Conversely, quantification of fluorescence intensity confirmed a high accumulation of mitochondria at the somite edges in Myomaker-depleted fish compared with controls (Figure 6E). These findings show that a complete myoblast fusion is required to spread mitochondria to the center of the somite and highlight the synchronization between cell maturation and mitochondria organization in the myotome (Figure 6F).

### Morphogenes Ensure Synchronization Between Organogenesis and Organellogenesis

**FIGURE 7:**
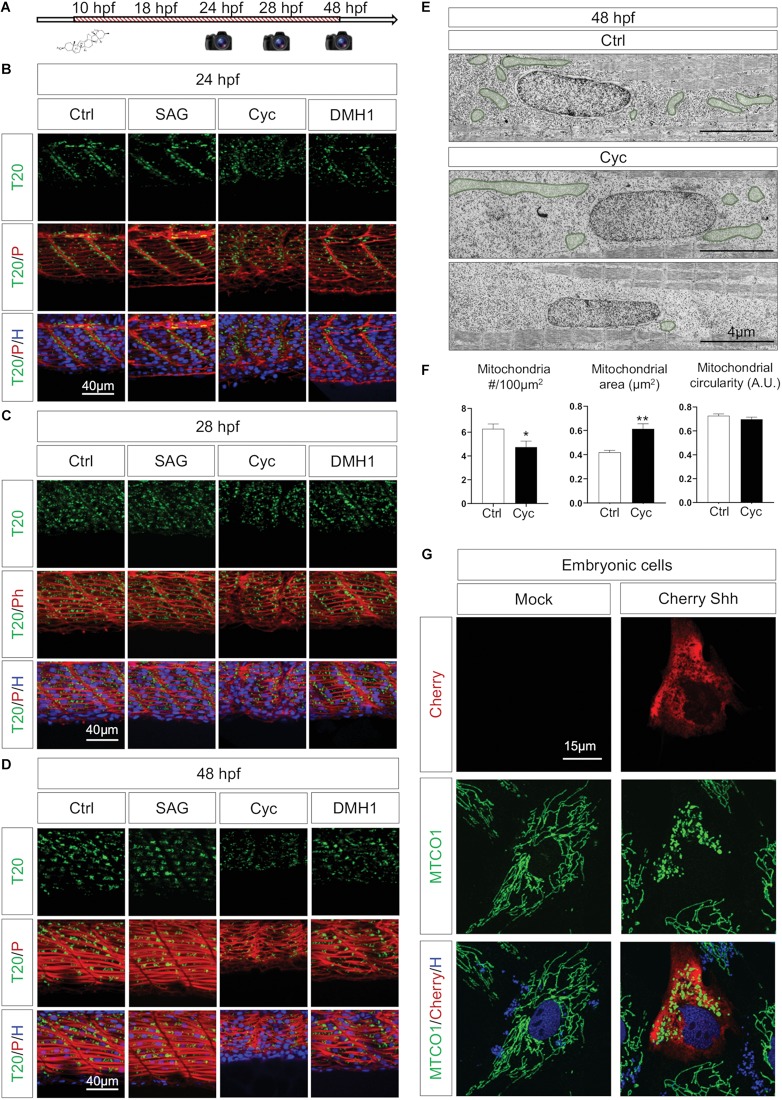
Shh signaling synchronizes tissue formation and mitochondria network maturation. **(A)** Pharmacological experiments design: agents were administered at 10 hpf, images captured at 24, 28, and 48 hpf. **(B–D)** Confocal imaging of mitochondrial network (Tomm20, T20; green) counterstained with Phalloidin (P; red) and Hoescht (H; blue) after administration of pharmacological agents to activate Shh signaling (SAG), inhibit Shh signaling (Cyclopamine, Cyc) or antagonize BMP signaling (DMH1) at 24 hpf **(B)**, 28 hpf **(C)**, and 48 hpf **(D)** in embryos submitted to the different drugs and in controls (Ctrl). **(E)** Electron micrographs of longitudinal sections at 48 hpf in Ctrl and Cyc treated embryo. Mitochondria are overlaid in green. **(F)** Quantification of mitochondria number, mean area and circularity at 48 hpf in Cyc treated embryos and Ctrl from longitudinal electron micrographs (*n* = 5 micrographs per group). **(G)** Confocal imaging of embryonic cells cultured from 10 hpf bud stage embryos either transfected with empty vector (Mock) or expressing Cherry-Shh. Mitochondria network is labeled with the component of complex IV MTCO1 (green), counterstained by Cherry (red) and Hoechst (H; blue). Bars are mean ± SEM. ^∗^*P* < 0.05, ^∗∗^*P* < 0.01, unpaired *T*-test. See Supplementary Figure S7.

Neither neuronal stimulation nor muscular contraction are responsible for mitochondrial organization in the myotome, but mitochondrial patterning is dependent on myoblast maturation, thus suggesting the involvement of morphogenes to ensure the synchronization between organogenesis and organellogenesis. Among other effects, such as defining the dorsoventral axis of somites (Ingham and Kim, 2005; Maurya et al., 2011; Aviles et al., 2013), Shh and BMP are known to impact myoblast identity (Lewis, 2006; Maurya et al., 2011). Shh signaling controls myogenesis through the activation of Myf5 and MyoD (Coutelle et al., 2001), regulates slow and fast-twitch fibers balance (Feng et al., 2006), activates genes cascades to develop and maintain slow fibers (Blagden et al., 1997; Lewis et al., 1999) as well as to promote growth and elongation of fast fibers (Henry and Amacher, 2004; Feng et al., 2006). We pursued to question whether BMP and Shh impact *in vivo* mitochondria organization using pharmacological experiments from bud stage (10 hpf). The administration of 10 μM DMH1, a BMP specific inhibitor (Hao et al., 2010) does not affect mitochondrial network in the myotomes despite muscle atrophy (Figures 7A–D) and morphological defects with shorter tails. Treatment with 10 μM of Smoothened Agonist (SAG), an activator of the Shh pathway, has no impact on mitochondrial network. Shh inhibition with 50 μM cyclopamine abolishes mitochondria stacking at somite boundaries in 24 hpf embryos (Figure 7B) and lowers mitochondrial signal at 48 hpf (Figure 7D). Upon quantification, cyclopamine treated animals present a reduction in mitochondria numbers with increases in their mean area (Figures 7E,F). These finding highlight the effect of Shh on mitochondria organization in embryos.

Shh effect can result from the alteration of the cellular network or, at the subcellular level, from a direct impact on mitochondria. Indeed, a direct effect of Shh on mitochondria had been previously observed in neuronal cultures in post-developmental conditions (Malhotra et al., 2016; Yao et al., 2017). To explore the potential role of Shh on mitochondria dynamics in embryonic cells, we cultured zebrafish cells from bud stage embryos (10 hpf) and transfected them with a plasmid expressing Cherry-tagged Shh (NM_131063.3) or an empty vector (Mock). The expression of Shh modifies the mitochondrial network, which appears fragmented (Figure 7G). Similarly, the direct activation of Shh pathway through the administration of Smoothened analog, SAG, induced a strong fragmentation of mitochondria in embryonic cells (Supplementary Figure S7). Altogether, these findings confirm that Shh modulates mitochondria dynamics at the subcellular level participating in the synchronization between muscle and organelle upon embryogenesis.

### Mitochondrial Quality Impacts Developmental Rhythm

**FIGURE 8:**
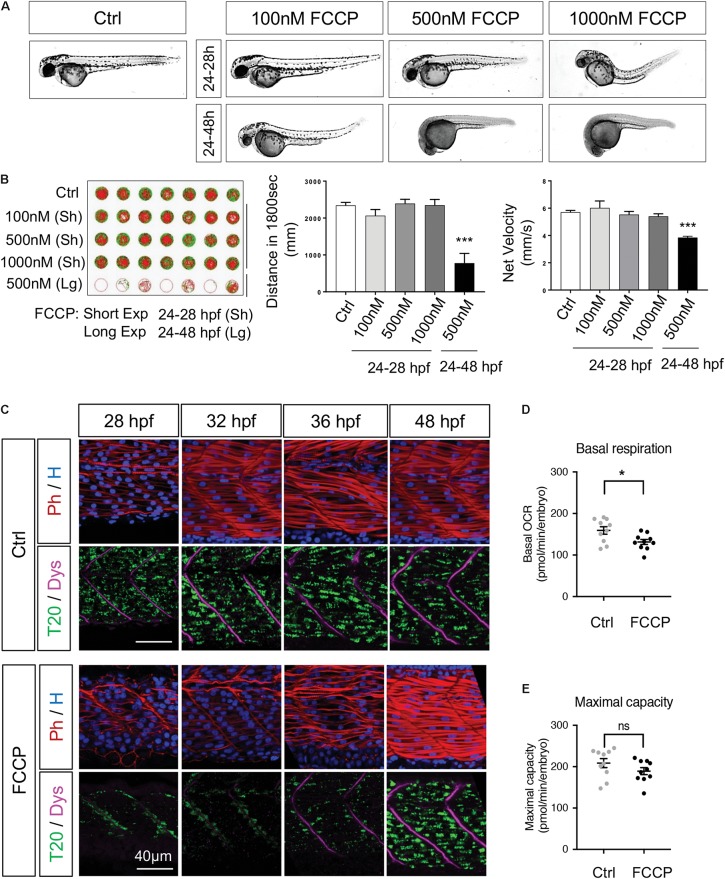
Mitochondrial quality is indispensable for embryogenesis. **(A)** Live embryos at 48 hpf after administration of FCCP with different doses and exposure durations. **(B)** Spontaneous locomotion in 5 dpf larvae after FCCP with different doses and durations. Slow velocity green lines are speeds between 3 and 6 mm/s. Fast velocity red lines are speeds > 6 mm/s (*n* = 12 fish per group). **(C)** Confocal imaging of mitochondrial network (Tomm20, T20; green) counterstained with Phalloidin (Ph; red), Dystrophin (Dys; magenta) and Hoescht (H; blue) with or without FCCP administration (500 nM from 24 to 28 hpf). **(D,E)** Basal respiration **(D)** and maximal respiration **(E)** in 48 hpf embryos with or without FCCP administration (500 nM from 24 to 28 hpf, *n* = 10 fish per group). Quantitative data are mean ± SEM. ^∗^*P* < 0.05, ^∗∗∗^*P* < 0.0001, unpaired *T*-test compared to control embryos (Ctrl). See also Supplementary Figure S8 and Supplementary Videos S2, S3.

Taken together, our results show that mitochondria dynamics follow precisely the key steps of embryogenesis, and the maturation of myotomes. This perfect synchronization raised the converse question: is mitochondrial quality, i.e., mitochondria membrane potential and ATP production, modulating organogenesis? To respond, we first uncoupled mitochondria with the use of FCCP, a ionophore known to abolish mitochondria membrane potential thus impacting ATP production. We administered FCCP in egg water with three doses (100, 500, and 1000 nM) and two exposure durations, ‘short’ from 24 to 28 hpf and ‘long’ from 24 to 48 hpf (Figure 8A). Short exposures show no impact on embryo morphology except the highest dose. Long exposures of 500 and 1000 nM stop development, maintaining living animals in developmental stand-by. Removing FCCP after the 500 nM long exposure triggers rebooting. Removing FCCP after the 1000 nM long exposure results in animal death. Lightsheet imaging after FCCP removal at 28 hpf revealed delayed mitochondria spreading (Supplementary Video S2, compared to control Supplementary Video S3) with a complete recovery of the mitochondrial network at 48 hpf (Figure 8C). These observations from short and long exposure to FCCP raised two questions. First, if this was a specific effect of FCCP thus if other known drugs affecting mitochondria had the same effect. The second question was related to the recovery capacity after the long exposure of FCCP thus to the resilience of embryogenesis progression after a transient loss of mitochondrial efficiency.

To understand if the delay in mitochondria spreading from 24 to 28 hpf was a particular action of FCCP, we first evaluated the impact of short exposures to other drugs known to have specific effects on mitochondria (Supplementary Figure S8A). The ionophore valinomycin has the same effect than FCCP on mitochondria patterning at 28 hpf. Exposure to rotenone and oligomycin, which inhibit respectively ETC CI and CV, from 24 to 28 hpf, show that alterations of these ETC complexes result in similar arrest of mitochondria patterning. Hydrogen peroxide (H_2_O_2_) exposure has no effect on mitochondria spreading thus pointing to the fact that oxidative stress does not affect mitochondrial pattern at 28 hpf. Similarly to the short exposure, a long exposure to oligomycin from 24 to 48 hpf confirms the same impact as FCCP with an arrest of development (Supplementary Figure S8B). The chemical depletion of water oxygen by Na_2_SO_3_ results in the equivalent inhibition of embryogenesis progression. Removing FCCP, oligomycin or Na_2_SO_3_ at 48 hpf restores development with a survival rate at 5 dpf going from 24% for oligomycin to 70% for Na_2_SO_3_. H_2_O_2_ and Paraquat do not affect developmental rate or survival (Supplementary Figures S8B,C). Taken together, these results show that a large array of alterations known to impact mitochondrial function, going from depletion of water oxygen, disturbances of the proton gradient or inhibition of selected ETC complexes, converge in a reversible arrest of embryogenesis. These findings unveil that an efficient mitochondrial pool is needed to drive the proper timing of embryogenesis.

To explore the resilience after mitochondrial altered patterning, we followed embryos submitted to long term FCCP at the end of embryogenesis and the larval stage. At 5 dpf, embryos submitted to the short protocols present no impairment in swimming distance or velocity (Figure 8B) while embryos submitted to the 500 nM long exposure display impaired swimming due to their delayed development. Functional analyses indicate similar maximal respiration at 48 hpf but lower basal respiration in FCCP treated embryos (Figures 8D,E). These observations confirm the global recovery of mitochondria network at 48 hpf. The transient effect of FCCP administered from 24 to 28 hpf shows the importance of mitochondria function for embryogenesis, particularly on the synchrony between organellogenesis and organogenesis.

## Discussion

While previous studies unveiled extensive changes of mitochondria during the first steps of embryogenesis, the adaptation of these organelles to face organ maturation remains unknown (Bavister and Squirrell, 2000; Squirrell et al., 2003; Lima et al., 2018). Our findings reveal the existence of three distinct mitochondrial patterns of distribution during somitogenesis. Formally, we successively witness (1) a homogeneous distribution of mitochondria in myotomes during early embryogenesis (18 hpf), followed by (2) a localized distribution of mitochondria along the somite boundaries at 24 hpf, finally followed by the spreading of mitochondria across the myotome at 28 hpf which ends up in (3) a homogeneous distribution of mitochondria throughout the somite at 36 hpf. The latter pattern is then maintained until the end of embryogenesis, which in zebrafish corresponds to 48 hpf. Gene expression supports this three-step pattern with two peaks in markers of biogenesis, a progressive increase in fission, low levels of mitophagy markers and a constant presence of markers of active mitochondrial transport.

These findings show the synchronization between somite maturation and mitochondrial network maturation, thus between organogenesis and organellogenesis. This synchronization is not driven by neuronal activation or muscle contraction. Inhibition of Shh pathway from the beginning of bud stage affects mitochondria patterning, suggesting that Shh, a well-established factor of muscle development, may play a role as a conductor in this synchronization. The effects of Shh on mitochondrial network integrity can be a consequence of its control of cell fate (Barresi et al., 2000; Henry et al., 2005) or by a direct signal that regulates mitochondrial network configuration. In line with previous works that confirmed a direct action of Shh on mitochondrial dynamics (Malhotra et al., 2016; Yao et al., 2017; Kaushal et al., 2018) and oxidative capacity (Alam et al., 2016; Yao et al., 2017) in post-mitotic or cancer cells, we observe a direct effect of Shh on mitochondria in embryonic cells. Mechanisms underlying this direct effect seem variable depending on the cell type. In rat hippocampal neurons, Shh signaling reduced DRP1 and mitochondrial fragmentation, increasing mitochondrial mass, membrane potential and respiration (Yao et al., 2017). In lung cancer cells, inhibition of Shh signaling reduced respiration and induced mitochondrial fragmentation with recruitment of Drp1 to the outer membrane (Alam et al., 2016). Similarly, in endometrial hyperplasia cells, Shh inhibition induced mitochondria depolarization and fragmentation with increased expression of Drp1 (Kaushal et al., 2018). In a developmental context, in cerebellar granule neuron precursors, it was not inhibition but Shh treatment that induced the above-mentioned modifications. Due to the fact that Drp1 was not modified but that MFN1 and MFN2 were decreased, the authors concluded that the suppression of fusion proteins accounted for the mitochondria fragmented appearance in Shh-treated cells (Malhotra et al., 2016). Our results confirm a specific effect of Shh on mitochondria during development that may be necessary to face the challenge of cell maturation during embryogenesis.

The synchronization between cell and mitochondrial fate points to the co-dependence between tissue formation and organelle organization. Indeed, the time course of embryo development controls the dynamics of mitochondria evolution. Reciprocally, mitochondria features play a crucial role to determine the tempo of embryonic development. The absence of efficient mitochondrial ATP production does not lead to an immediate lethality, but holds in stand-by the developmental progression. Drugs known to alter the proton gradient, inhibit ETC complexes or that deplete water oxygen similarly result in an arrest of the developmental progression. Upon wash-out of these drugs, development continues, indicating that ATP availability from mitochondria set the pace of organ development. Our observations performed in the second part of embryogenesis, from 24 hpf onward, are in accordance with previous results suggesting that a blockage of mitochondrial ATP production through rotenone administration can interrupt early embryogenesis at 5 hpf (Byrnes et al., 2018). Other studies pointed out to the importance of functional ETC proteins in timely development (Baden et al., 2007; Flinn et al., 2009; Zurita Rendon et al., 2014; Byrnes et al., 2018). Thus, our data indicate that not only embryogenesis is linked with mitochondrial fate but the quality of respiration defines the progression of the developmental pattern.

In conclusion, our results highlight the synchronous spatio-temporal organization of mitochondria and somite development orchestrated by Shh pathway. The burst of biogenesis observed at the end of somitogenesis establishes the expansion of the mitochondrial mass from the maternal pool. Subsequent spreading of mitochondria goes parallel to myofiber maturation. Mitochondria quality is required to allow these correct timings in development, which may explain the observed stability of ETC complexes and SCs. This work underlines the importance of a coordinated dialogue between mitochondria and tissue maturation in post-gastrulation stages. These findings point to the importance of organellogenesis for vertebrate development and question the dynamics and role of other organelles in embryogenesis.

## Materials and Methods

### Zebrafish Husbandry and Strains

Zebrafish (*Danio rerio*, Oregon AB) were housed at the Zebrafish facility of the School of Biology and Medicine, maintained at 28.5°C and on a 14:10 h light dark. Embryos were staged by hours (h) or days (d) post fertilization according to Kimmel et al. (1995). To follow mitochondria through the reporter gene Tomm20, transgenic males actc1b:tomm20-ZsGreen (Line designation: nei007) were crossed with AB wild type females. Zebrafish husbandry and well fare were approved by the *Service de la consommation et des affaires vétérinaires* (SCAV) of the Canton of Vaud.

### Drug Administration

All the drugs were directly delivered in egg water of dechorionated embryos with the exception of αBungarotoxin as specified below. Mitochondria-targeting drugs (Figure 8 and Supplementary Figure S8) were added from 24 hpf and rinsed at 28 hpf or at 48 hpf, with the following concentrations: FCCP (C2920, Sigma-Aldrich, 100 nM, 500 nM, 1 μM); Oligomycin A (75351, Sigma-Aldrich, 3 μM); Valinomycin (V1644, Invitrogen, 1 μM); Rotenone (R8875, Sigma-Adrich, 100 nM); H_2_O_2_ (31642, Sigma-Aldrich, 100 μM, 2 mM); MDIVI-1 (M0199, Sigma-Aldrich, 50 μM); Paraquat (36541, Sigma-Aldrich, 100 μM) and Na_2_SO_3_ (S0505, Sigma-Aldrich, 150 mM). Morphogens-targeting drugs (Figure 7) were applied from 10 hpf: Shh-antagonist Cyclopamine (C4116, Sigma-Aldrich, 50 μM); Shh-agonist SAG (sml1314; Sigma-Aldrich, 10 μM), BMP-antagonist DMH1 (D8946, Sigma-Aldrich, 10 μM). To block muscle contractions, 4 nl of αBungarotoxin-TRITC (T0195, Sigma-Aldrich, 100 μM) were injected in the blood stream of 24 hpf embryos.

### Morpholino and mRNA Microinjection

Myomaker antisense Morpholino oligonucleotides (Gene Tools) were designed from Landemaine et al. (2014) and microinjected into one-cell stage embryos accordingly to standard protocols. A 1nl volume was injected for a final amount of 0.5 pmol.

DRP1-K38A mRNA was synthetized using the plasmid pcDNA3-Drp1K38A [45161, Addgene (Smirnova et al., 1998)] linearized with Not1 and transcribed by mMESSAGE mMACHINE T7 Transcription Kit (AM1344, Thermo Fisher Scientific). Five hundred pg of mRNA were injected into one-cell stage embryos with a volume of 1 nl.

### Electrical Pulse Stimulation

Three dechorionated embryos were placed in each well of a six well plate with 2 ml of egg water and were submitted to 2.0 ms electrical pulse trains (C-Pace EM, IonOptix, Dublin, Ireland). Specific stimulation protocols were created to achieve natural contraction frequency (Saint-Amant and Drapeau, 1998; Hirata et al., 2009) with 40 V and 3 Hz from 20 to 24 hpf and with 40 V and 1 Hz from 24 to 28 hpf (Figure 5).

### Locomotion Assays

Zebrafish spontaneous activity was monitored at 5 dpf using a Zebrabox recording system (Viewpoint, Lyon, France). One fish was placed per well on a 96 well plate. Recording was performed during 30 min in 0% of luminosity after a 25% of luminosity for 30 min.

### RNA Extraction and qPCR

Total RNA was extracted from 50–100 zebrafish embryos or dissected somites at the developmental stages specified in the text and from adult muscle (male, 6 months) using TRIzol Reagent (15596026, Invitrogen), following the procedure in Peterson and Freeman (2009). CDNA was synthesized by reverse transcription (RT) using both Oligo(dT) (18418020, Invitrogen) and Random Hexamers (N8080127, Invitrogen) with Super Script II Reverse Transcriptase (18064014, Invitrogen) based on standard protocols. Each RT product was mixed with Power SYBR Green PCR Master Mix (4367659, Thermo Fischer Scientific) and with 300 nM of forward and reverse primers (Supplementary Table S1 and Figure 4). Reaction mix was cycled on StepOnePlus (Applied Biosystems, Foster City, CA, United States). Relative expression of mRNA was estimated using the 2−ΔΔCT method using 18S rRNA as reference.

### Immunolabelling and Staining

Immunostaining was performed on whole mounted embryos from 18 to 48 hpf as specified. After anesthesia with tricaine (MS-222, Sigma-Aldrich), embryos were fixed in 4%PFA for 2 h prior to permeabilization in PBS/Triton1% for 2 h. Primary antibodies were applied overnight at 4°C at specific concentrations: anti-Synaptotagmin2 (ZNP1, DSHB, 1/100); anti-Dystrophin (MANDRA1, DSHB, 1/50); anti-MTCO1 (ab14705, Abcam, 1/300); anti-light meromyosin portion of heavy chain myosin II (MF20, DSHB, 1/20). Subsequent labeling were applied as needed for 1 h at room temperature: Phalloidin-TRICT (P1951, Sigma-Aldrich, 100 nM) and αBungarotoxin-TRITC (T0195, Sigma-Aldrich, 100 nM).

### Confocal and Lightsheet Imaging

Image acquisition for fixed samples was performed on a confocal laser scanning microscope (LSM510, Zeiss, Stockholm, Sweden) at the Cell Imaging Facility. All the pictures correspond to one confocal plane. 3D videos of fixed embryos (Supplementary Videos S2, S3) were performed on a Lighsheet Fluorescent microscope (Z1, Zeiss) at the Bioimaging and Optics platform from the EPFL. The time lapse movie on anesthetized embryos from 20 to 40 hpf (Supplementary Video S1) was recorded using a prototype of double illumination inverted light sheet microscope hosted in the lab of Prof. Andrew Oates (School of Life Sciences, EPFL, Lausanne). This light sheet microscope is now commercialized under the name of *ILS1 Live* by the EPFL start-up company Viventis Microscopy Sàrl.

### Electron Microscopy

Two consecutive fixations were performed in PB 0.1M/Formaldehyde 4%/Glutaraldehyde 2.5% at room temperature for 2 h. The first one in whole fish and the second one after cutting head and tail with a razor blade to enhance penetration. Fish older than 24 hpf were pre-treated with 1-phenyl-2-thiourea (P7629, Sigma-Aldrich, 75 μM) to inhibit pigmentation (Karlsson et al., 2001). The samples were then washed and treated with osmium (PB 0.1M/osmium tetroxide 1%/potassium ferrocyanide 1.5%) for 2 h and stored in clear water. Embryo were cut longitudinally on a Leica Ultracut (Leica Mikrosysteme GmbH, Vienna, Austria) and pictures were taken around the 16th somite from the head with a transmission electron microscope Philips CM100 (Thermo Fisher Scientific, Waltham, MA, United States) at the Electron Microscopy Facility.

### Image Analyses and Quantifications

Mitochondria were manually traced in non-overlapping electron micrographs sections of 200 × 200 μm^2^. Mitochondria number, area and shape were quantified with ImageJ ROI manager (NIH, Bethesda, MD, United States). Mean area was obtained as the mean surface of individual mitochondria in a given micrograph. The circularity index was determined with the formula 4π(area/perimeter^2^). A value of 1 corresponds to a circle.

Mitochondria spread through the somite was estimated by the ratio of Tomm20-zGreen fluorescence intensity from the confocal pictures. Fluorescence intensity was determined on equal areas along boundaries and in the center of somites.

### Mitochondria Extracts and Blue Native Gels

Mitochondria isolation was performed from dechorionated and deyolked embryos from 18 to 48 hpf, 5 dpf embryos and adult muscle using an established protocol (Jha et al., 2016) slightly modified as follow. First homogenization was performed in 2 ml cold isolation buffer (IB) at 1500 rpm after 30 strokes. The homogenized extracts were then centrifuged three times at 600 *g* for 10 min at 4°C in order to remove cellular debris. The mitochondrial fraction was pelleted at 10000 *g* for 10 min at 4°C and subsequently washed. The mitochondrial pellet was suspended in cold IB and stored at −80°C in 10 μg aliquots. A total of 10ug mitochondrial extracts were submitted to digitonin to allow their solubilization at the final concentration of 8 g/g protein. After 30 min in ice, the supernatant was loaded into 4–13% BN gels, run and transferred in PVDF membrane (10600021, Amersham Hybond). The membrane was cut and incubated with primary antibodies against CI (NDUFS3, Abcam, ab14711, 1/500), CII (SDHB, Abcam, ab14714, 1/1000), CIII (UQCRC2, Proteintech, 14742-1-AP, 1/1000), CIV (MTCO1, Abcam, ab14705, 1/1000) and CV (ATP5A, Abcam, ab14748, 1/1000). Mouse and rabbit Western Breeze Chromogenic Immunodetection kits were used (Thermofisher Scientific).

### Embryonic Cells Culture and Transfection

Bud stage embryos (10 hpf) were digested in trypsin-EDTA and cultured at 28°C in L15 complete culture medium as described in Vallone et al. (2007). After 1 day, cells were transfected with lipofectamine 2000 (11668-027, Thermofisher Scientific). mRNA Shh (ShhA, NM_131063) was cloned from *Danio rerio* embryonic cDNA and expressed in a pcDNA-Cherry Gateway vector (Thermo Fisher Scientific). Mock transfection was done with the empty plasmid.

### Whole Embryo Oxygen Consumption Rate

Mitochondrial function was determined with a Seahorse XFe24 extracellular flux analyzer (Seahorse Bioscience, Billerica, MA, United States). Oxygen consumption rate (OCR) was measured in dechorionated embryos at 48 hpf. Embryos were placed one per well on an islet capture microplate filled with E3 medium. The plate was incubated without CO_2_ at 28°C for 30 min. OCR was measured at baseline as an indication for basal respiration and then measured after an injection of 2 μM of FCCP to determine maximal respiration. Finally, 0.5 μM of Antimycin A and 0.5 μM of Rotenone were added to block CIII and CI respectively.

## Data Availability Statement

No genetic, genomic, RNA sequencing, proteomics, or any other -omics datasets were generated for this study. The raw data supporting the conclusions of this manuscript will be made available by the authors, without undue reservation, to any qualified researcher.

## Ethics Statement

The animal facility is approved by the *Service de la consommation et des affaires vétérinaires (SCAV)* of the Canton of Vaud.

## Author Contributions

YA, DG, SL, and FA contributed to the conception and design of the study. JR and PG created the transgenic reporter model. YA, DG, SL, and MG performed the experiments. YA, DG, SL, and FA organized the data, performed the statistical analyses, and created the figures. YA, DG, and SL wrote sections of the first draft. FA wrote the manuscript. All authors contributed to the manuscript revision, read and approved the submitted version.

## Conflict of Interest

PG and JR are employees of the Nestlé Institute of Health Sciences SA. The remaining authors declare that the research was conducted in the absence of any commercial or financial relationships that could be construed as a potential conflict of interest.
