# Supplementary material for: Mitochondria in Embryogenesis: An Organellogenesis Perspective
Source: Front Cell Dev Biol. 2019 Nov 22;7:282. doi: 10.3389/fcell.2019.00282 (PMC6883342; doi:10.3389/fcell.2019.00282)
Supplement: Supplementary file 1 [file Table_1.pdf]

Table S1, List of primers

Mitochondrial function	Gene Name	abbreviation	Primer	RT QPCR Primer sequence (5'-->3')	Origin
Housekeeping	18s rRNA	18s rRNA	18s rRNA (F)	AGCGTGCGGGAAACCACGAG	Artuso et al
			18s rRNA (R)	AAGCCGCAGGCTCCACTCCT	Artuso et al
Biogenesis	tfam	tfam	Tfam (F)	GCGAAAGATTGCCCAGCAGT	Artuso et al
			Tfam (R)	TTGTCGTTTTTCCTCCGCAAA	Artuso et al
	pgc1α	pgc1α	PGC1α (F)	GCGAGGGAACGAGTGGATTT	Parisi et al
			PGC1α (R)	CTCTCCACACCGAATCCTGA	Parisi et al
Fission	dnm1a	dnm1a	DNM1a (F)	CCGTGTCGCCCGCTAAC	Primer Express
			DNM1a (R)	CTTCATCCATCAGATCCAGTTTAGTG	Primer Express
Fusion	mfn2	mfn2	MFN2 (F)	CTCTCGACTGCAAGAGCAAAATC	Primer Express
			MFN2 (R)	CACGACTGGTGAAGGATGGA	Primer Express
Mitophagy	nip3a	nip3a	NIP3a (F)	AATGAAGGATTCAGACCGCATGGC	Feng et al
			NIP3a (R)	TGGCTCCAGTCTTCCTCATGCTAA	Feng et al
	pink1	pink1	PINK1 (F)	ACAGTGTACCGGGCCTTCAC	Primer Express
			PINK1 (R)	GAACAGTGTACGGTTACTGCCTAAAC	Primer Express
	park2	park2	Park2 (F)	CTTCTCCCAAACCGGATTCA	Primer Express
			Park2 (R)	CGTGTGTTGGGCATGATCAG	Primer Express
Transport	miro1	miro1	Miro1 (F)	GCCATCACAGTCACCAGGAA	Primer Express
			Miro1 (R)	AGCCTGCAGAAAGCCACTTTT	Primer Express
Transport	miro2	miro2	Miro2 (F)	CCGCTGACAGACGACTGCTA	Primer Express
			Miro2 (R)	GTCTGACACTCTTGCTCCATCAGTA	Primer Express

References:

Artuso, L., Romano, A., Verri, T., Domenichini, A., Argenton, F., Santorelli, F. M., et al. (2012). Mitochondrial DNA metabolism in early development of zebrafish (Danio rerio). Biochim. Biophys. Acta 1817, 1002–1011. doi: 10.1016/j.bbabio.2012.03.019

Feng, X., Liu, X., Zhang, W., and Xiao, W. (2011). p53 directly suppresses BNIP3 expression to protect against hypoxia-induced cell death. EMBO J. 30, 3397–3415. doi: 10.1038/emboj.2011.248

Parisi, A., Blattmann, P., Lizzo, G., Stutz, V., Strohm, L., Richard, J., et al. (2018). PGC1a and Exercise Adaptations in Zebrafish. bioRxiv. [preprint]. doi: 10.1101/483784
