# Supplementary material for: Mitochondria in Embryogenesis: An Organellogenesis Perspective
Source: Front Cell Dev Biol. 2019 Nov 22;7:282. doi: 10.3389/fcell.2019.00282 (PMC6883342; doi:10.3389/fcell.2019.00282)
Supplement: Supplementary file 5 [file Presentation_1.pdf]

Supplementary Material

Mitochondria in Embryogenesis: An Organellogenesis Perspective

Yoan Arribat, Dogan Grepper, Sylviane Lagarrigue, Joy Richard, Mélanie Gachet, Philipp Gut and Francesca Amati

Supplementary material includes three videos, eight figures and one table

Videos titles and legends

Video 1, Mitochondrial network in live zebrafish embryos.
Live imaging in embryos from 20 hpf to 40 hpf reveals mitochondrial network patterns of change (Tomm20; green). Acquisition corresponds to 1-Z plan. This time lapse movie was recorded using a prototype of double illumination inverted light sheet microscope (ILS1 Live, EPFL start-up company Viventis Microscopy Sàrl).

Video 2, Pharmacological alterations of mitochondrial pattern of change at 28hpf – Influence of FCCP.
Lightsheet 3D reconstitution of 28 hpf zebrafish embryos submitted to 500nM FCCP from 24 hpf. Staining corresponds to mitochondrial network Tomm20 (green) counterstained with Phalloidin (red) and Hoescht (blue).

Video 3, Mitochondrial pattern of change at 28hpf – control conditions.
Lightsheet 3D reconstitution of 28 hpf zebrafish embryo. Staining corresponds to mitochondrial network Tomm20 (green) counterstained with Phalloidin (red) and Hoescht (blue).
