# Supplementary figures and images for: Mitochondria in Embryogenesis: An Organellogenesis Perspective
Source: Front Cell Dev Biol. 2019 Nov 22;7:282. doi: 10.3389/fcell.2019.00282 (PMC6883342; doi:10.3389/fcell.2019.00282)

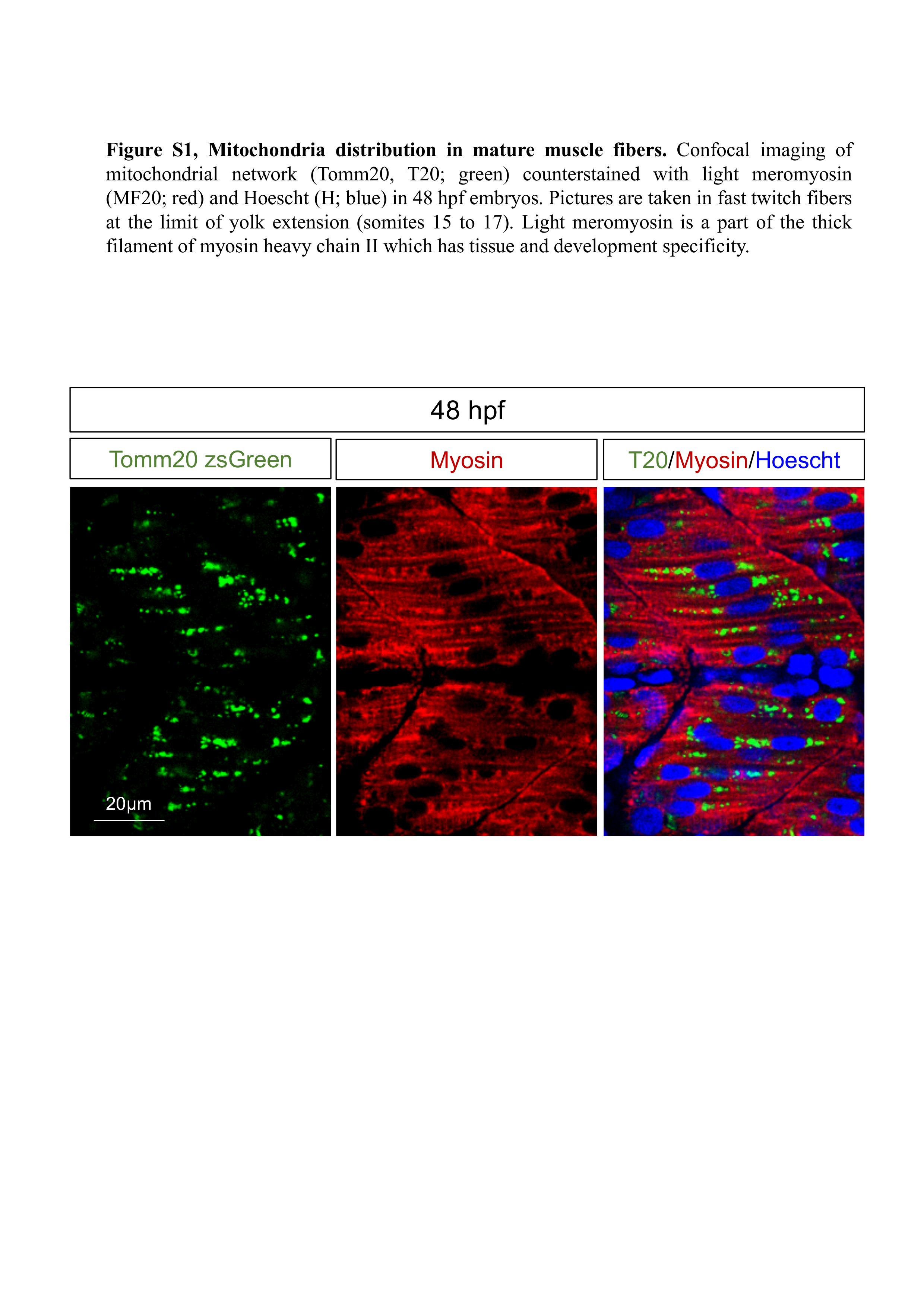

Supplement: Supplementary file 6 [file Image_1.tiff]

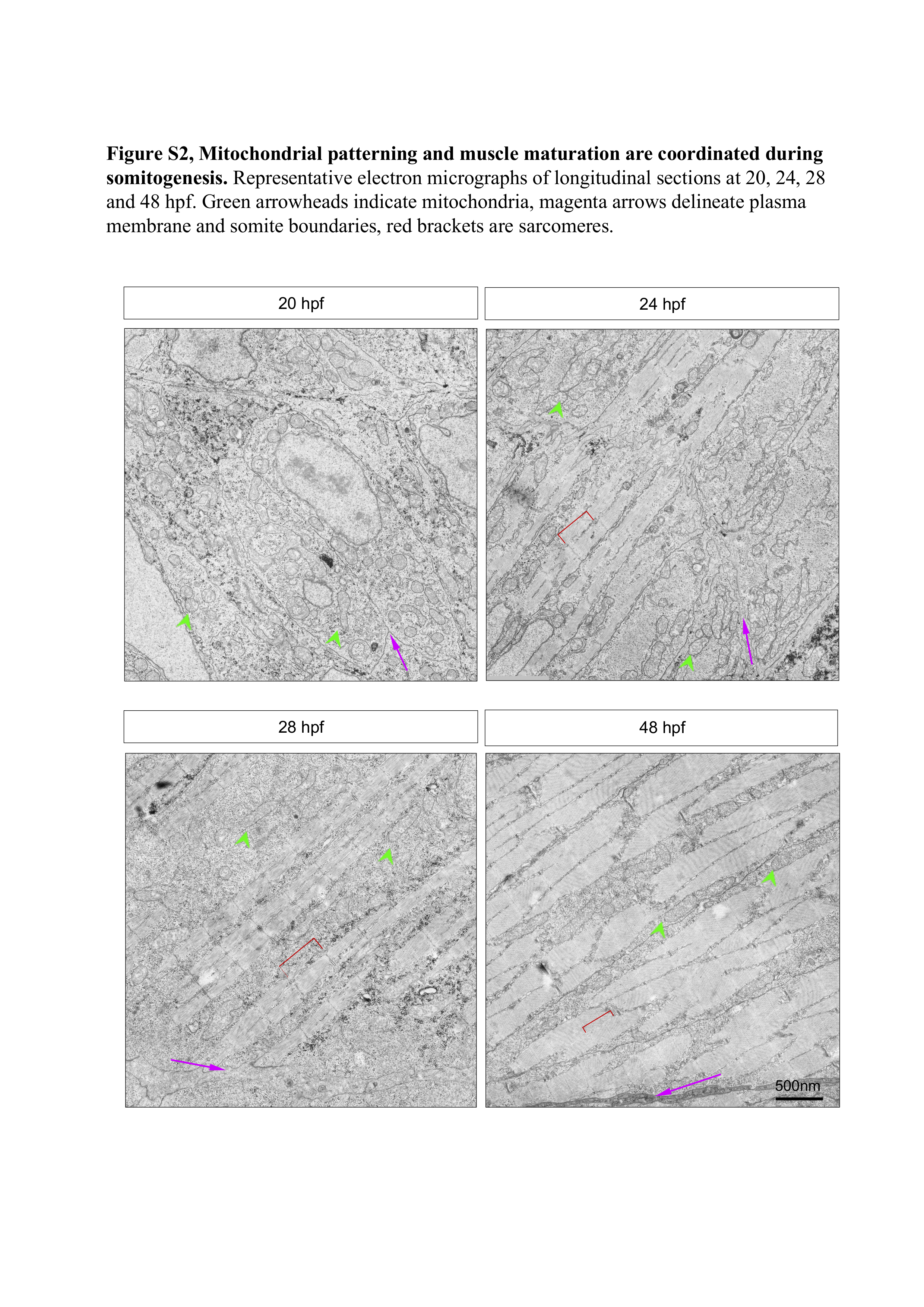

Supplement: Supplementary file 7 [file Image_2.tiff]

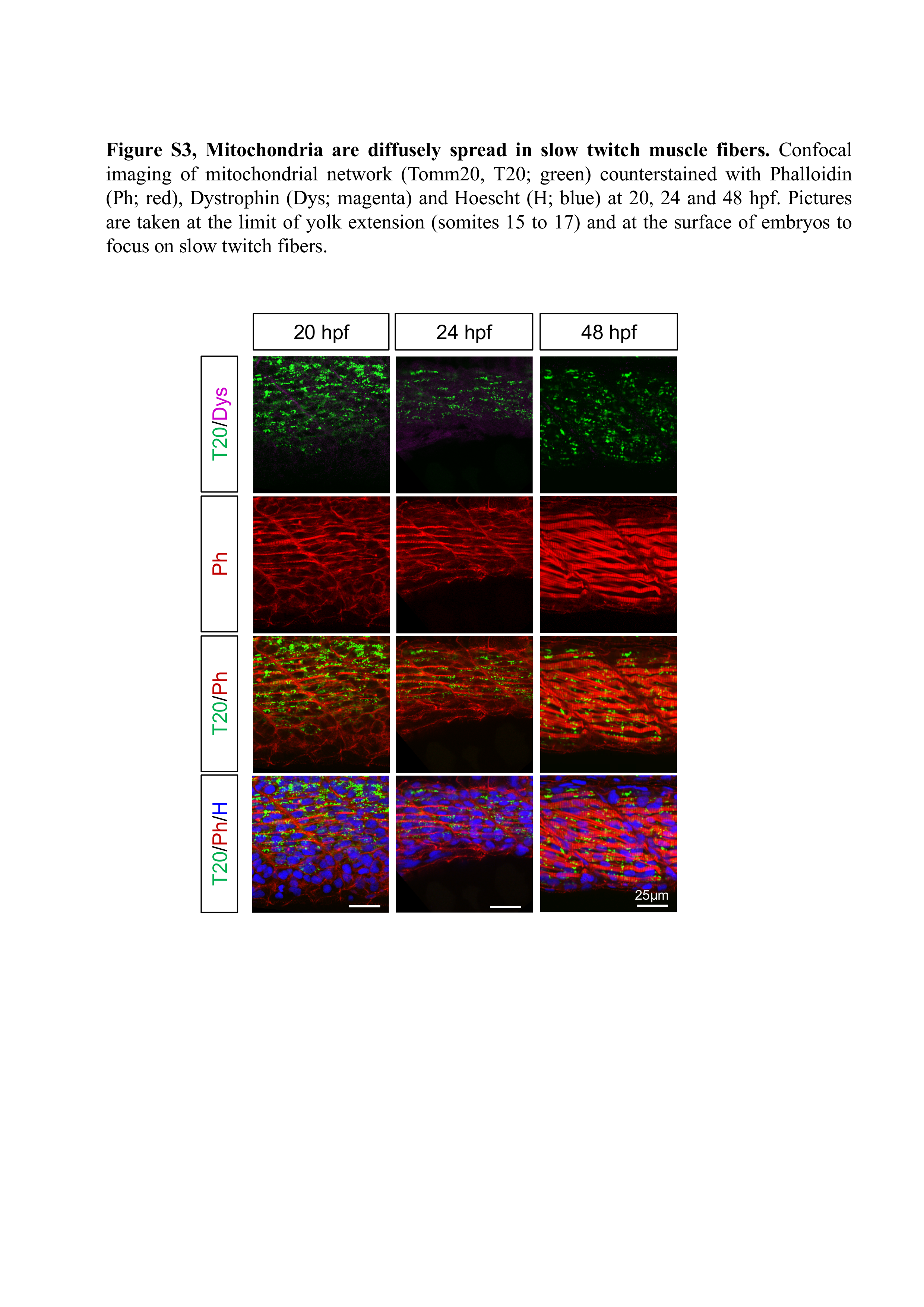

Supplement: Supplementary file 8 [file Image_3.tiff]

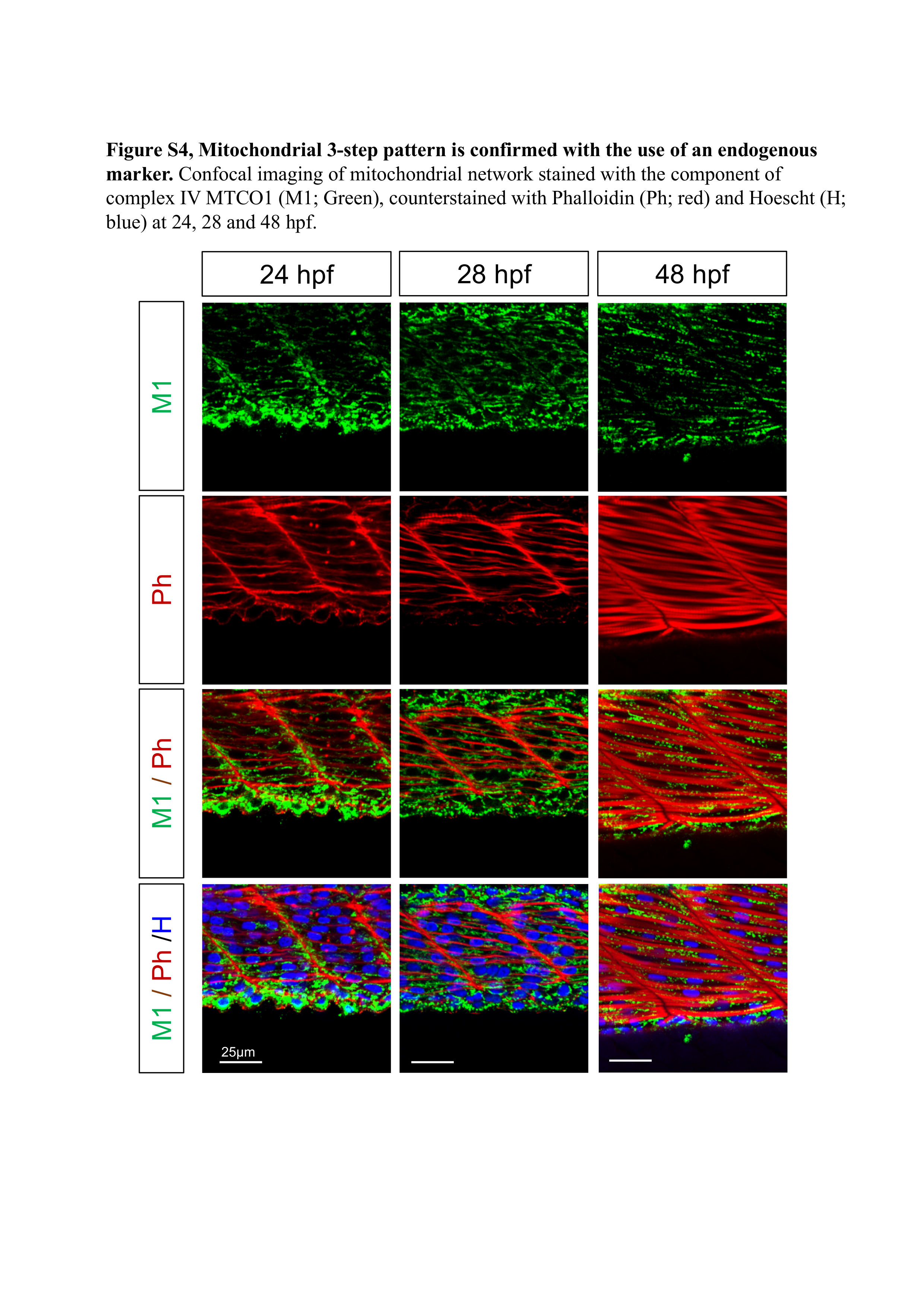

Supplement: Supplementary file 9 [file Image_4.tiff]

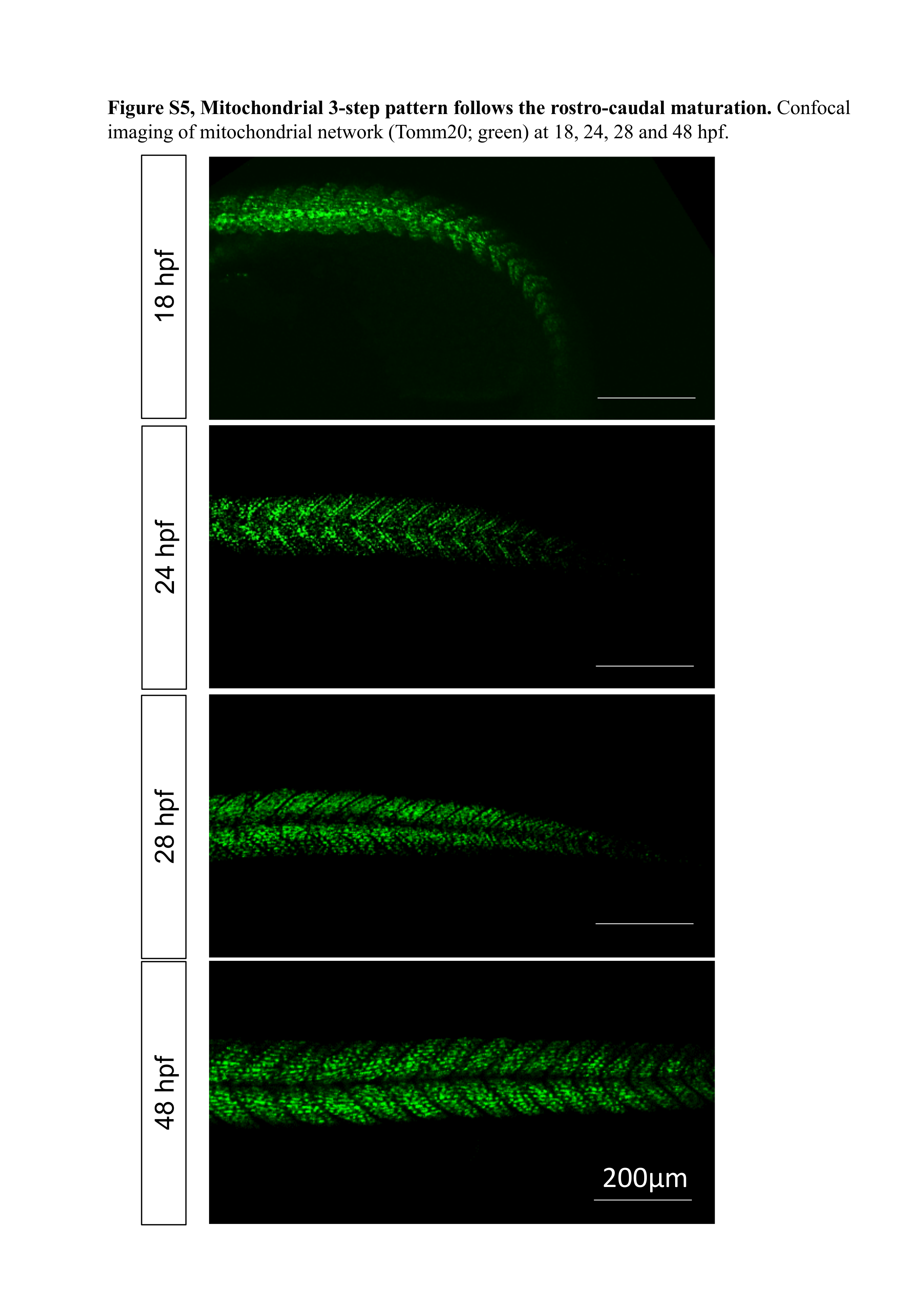

Supplement: Supplementary file 10 [file Image_5.tiff]

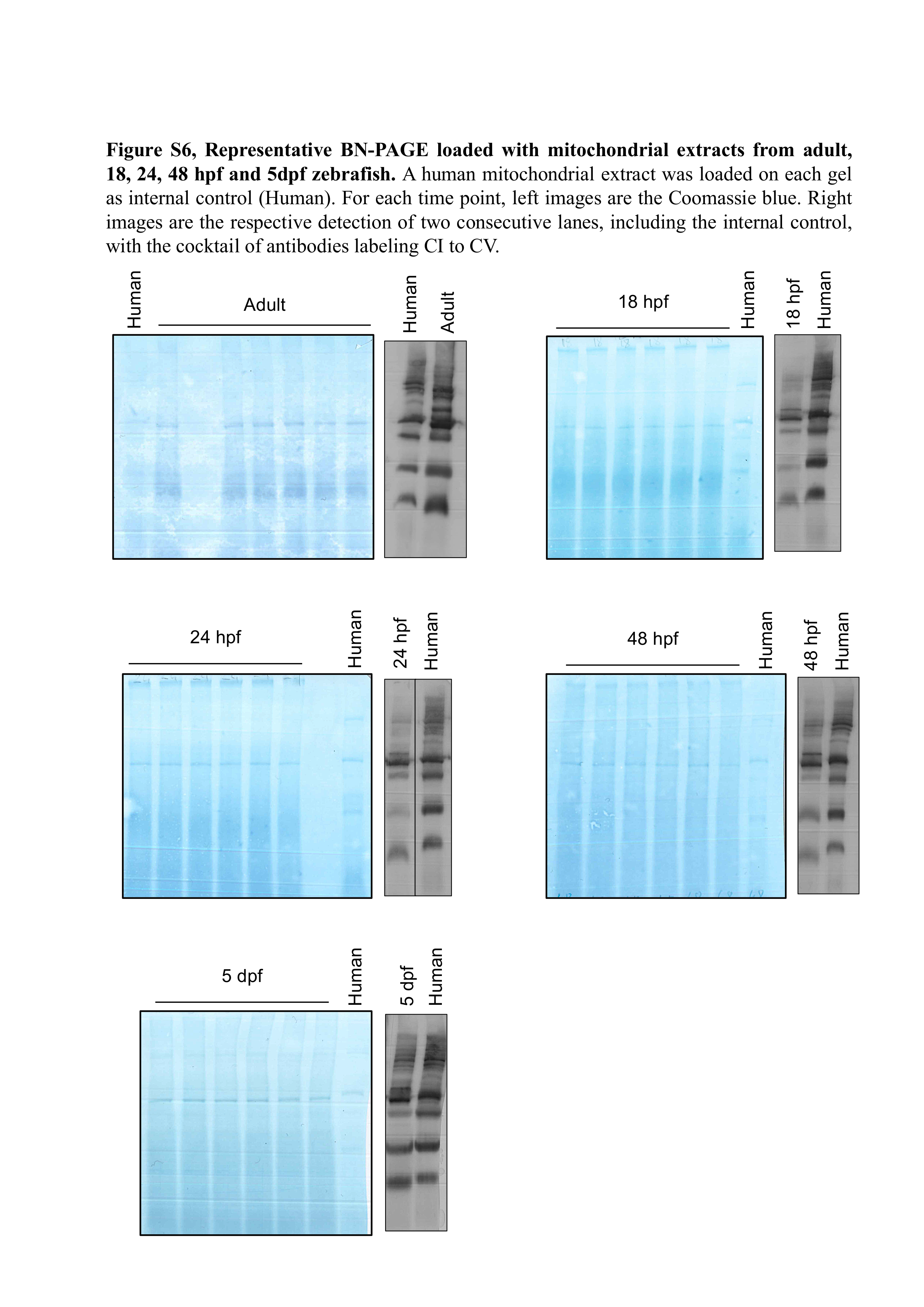

Supplement: Supplementary file 11 [file Image_6.tiff]

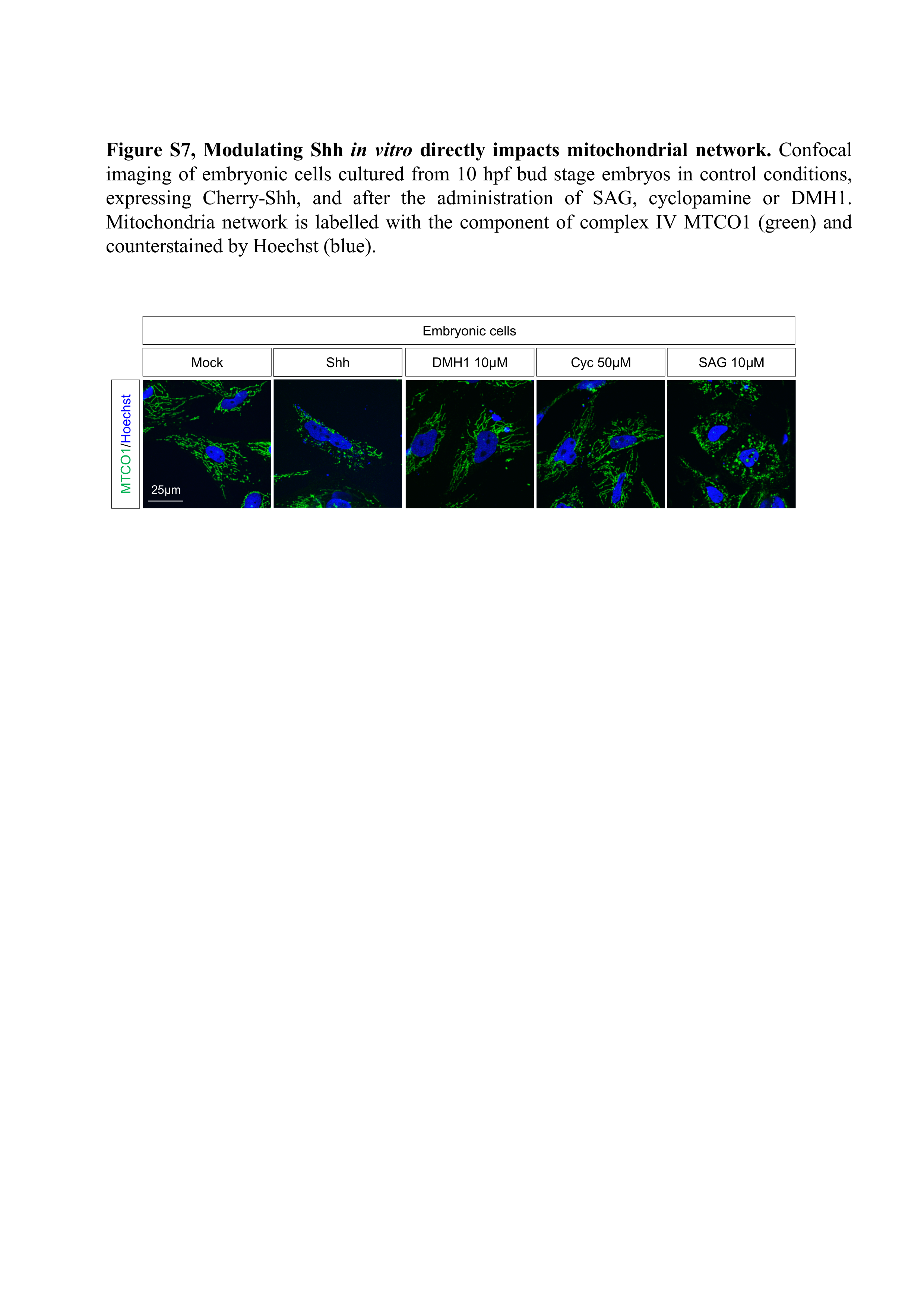

Supplement: Supplementary file 12 [file Image_7.tiff]

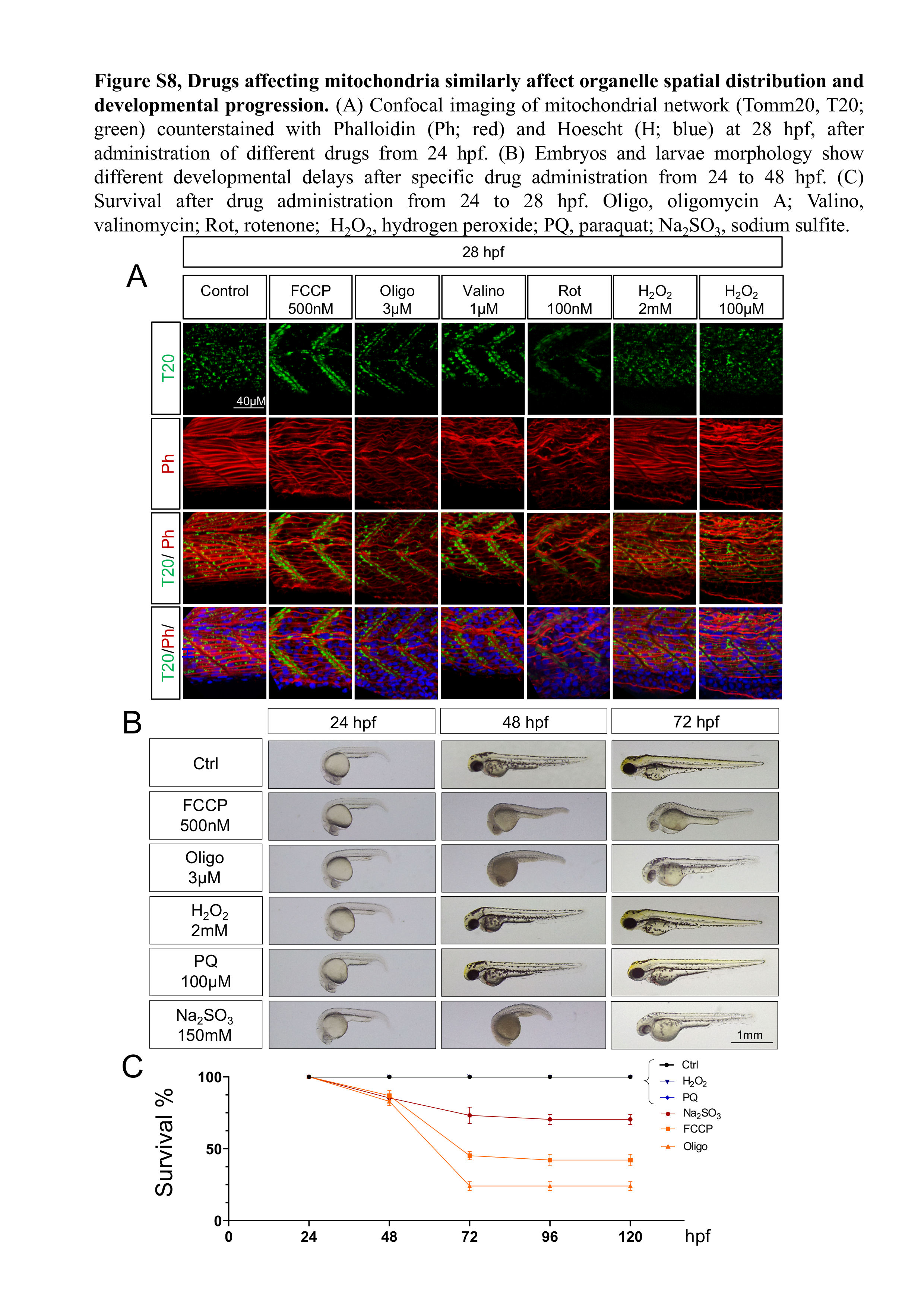

Supplement: Supplementary file 13 [file Image_8.tiff]
